# Multivariate pattern analysis of brain structure predicts functional outcome after auditory-based cognitive training interventions

**DOI:** 10.1038/s41537-021-00165-0

**Published:** 2021-08-19

**Authors:** Lana Kambeitz-Ilankovic, Sophia Vinogradov, Julian Wenzel, Melissa Fisher, Shalaila S. Haas, Linda Betz, Nora Penzel, Srikantan Nagarajan, Nikolaos Koutsouleris, Karuna Subramaniam

**Affiliations:** 1 Faculty of Medicine and University Hospital of Cologne, University of Cologne, Cologne, Germany; 2 Department of Psychiatry and Psychotherapy, Ludwig-Maximilian-University, Munich, Germany; 3 Department of Psychiatry, University of Minnesota, Minneapolis, MN USA; 4 Department of Psychiatry, Icahn School of Medicine at Mount Sinai, New York, NY USA; 5 Department of Basic Medical Sciences, Neuroscience and Sense Organs – University of Bari Aldo Moro, Bari, Italy; 6 Department of Radiology and Biomedical Imaging, University of California San Francisco, San Francisco, CA USA; 7 Institute of Psychiatry, Psychology and Neuroscience, King’s College London, London, UK; 8 Max Planck Institute of Psychiatry, Munich, Germany; 9 Department of Psychiatry, University of California San Francisco, San Francisco, CA USA

**Keywords:** Schizophrenia, Neuroscience

## Abstract

Cognitive gains following cognitive training interventions are associated with improved functioning in people with schizophrenia (SCZ). However, considerable inter-individual variability is observed. Here, we evaluate the sensitivity of brain structural features to predict functional response to auditory-based cognitive training (ABCT) at a single-subject level. We employed whole-brain multivariate pattern analysis with support vector machine (SVM) modeling to identify gray matter (GM) patterns that predicted higher vs. lower functioning after 40 h of ABCT at the single-subject level in SCZ patients. The generalization capacity of the SVM model was evaluated by applying the original model through an out-of-sample cross-validation analysis to unseen SCZ patients from an independent validation sample who underwent 50 h of ABCT. The whole-brain GM volume-based pattern classification predicted higher vs. lower functioning at follow-up with a balanced accuracy (BAC) of 69.4% (sensitivity 72.2%, specificity 66.7%) as determined by nested cross-validation. The neuroanatomical model was generalizable to an independent cohort with a BAC of 62.1% (sensitivity 90.9%, specificity 33.3%). In particular, greater baseline GM volumes in regions within superior temporal gyrus, thalamus, anterior cingulate, and cerebellum predicted improved functioning at the single-subject level following ABCT in SCZ participants. The present findings provide a structural MRI fingerprint associated with preserved GM volumes at a single baseline timepoint, which predicted improved functioning following an ABCT intervention, and serve as a model for how to facilitate precision clinical therapies for SCZ based on imaging data, operating at the single-subject level.

## Introduction

Occupational and social functioning are impaired in patients with schizophrenia (SCZ) and are associated with a range of neural system and clinical impairments^1–3^. Cognitive training (CT) interventions can drive neural system changes^4,5^ that are in turn associated with functional improvement^6–8^. Previous studies have shown training-induced restoration of neural activation patterns in the medial prefrontal cortex and anterior cingulate cortex (mPFC/ACC) that were associated with improved performance on a reality-monitoring task^4,9,10^, and which in turn predicted durable gains in real-world social functioning 6 months later. We have also shown enhanced activation in the dorsal lateral prefrontal cortex, which was associated with improved performance on a working memory task, and predicted better occupational functioning at 6-month follow-up^11^.

Although these findings are promising at the group level, it is clear that there is a large amount of inter-individual variability in neural systems and functional response to various forms of CT. Previous group-based studies have shown that baseline structural anatomical integrity^12–14^ is associated with greater responsiveness to CT, suggesting that certain individual neurobiological characteristics might determine who will benefit most from CT interventions, but individual-level predictions have not yet been demonstrated. In particular, prior research indicates that patients with SCZ show most prominent deficits in auditory processing, which contributed to higher-level cognitive impairments and poor functioning^15–17^. Promisingly, we have also found that the most prominent gains in auditory/verbal functions were induced after auditory-based CT (ABCT) interventions^18–22^. This work prompted us to investigate multivariate pattern analyses (MVPA) to identify baseline patterns in gray matter (GM) volume in patients with SCZ that predicted improved functioning after an ABCT intervention, operating at the single-subject level. Multivariate analyses of neuroanatomical brain properties have revealed high specificity of predicting improved functioning in clinical high-risk individuals with psychosis in single-site studies at the individual level^23,24^, and have also shown remarkable multisite generalizability^25^. However, no study has yet examined the critical question of which structural features at baseline most predict responsiveness to ABCT in chronically-ill SCZ, in terms of improving real-world functioning at the single-subject level.

Group-based studies have shown that greater GM volume has been predictive of improved functioning in SCZ patients and also associated with stronger resilience to functional deterioration^1,12^. Informed by these prior group-based studies and meta-analyses^1,12,13^, we hypothesized that patients who had greater GM volumes at baseline, particularly in prefrontal, thalamic, and temporal regions^1,12–14^, would show improved functioning at the single-subject level in response to 40 h of ABCT. We further explored the relationship between the decision values of the functioning classifier generated in SCZ patients and their clinical characteristics at baseline and at follow-up to test whether clinical symptom severity or medication dose were associated with individual level of functioning after 40 h of CT. Finally, we investigated whether our original GM machine learning model was sufficiently generalizable to an independent training cohort who also underwent ABCT.

## Results

### Participant characteristics

Table 1 summarizes the sociodemographic, clinical, and cognitive characteristics of the two study samples, separated by their Global Assessment of Functioning (GAF) score median split at the post-training timepoint. No significant differences with respect to age, years of education, illness duration, and antipsychotic medication dosage (chlorpromazine equivalents) at baseline were found between lower and higher functioning SCZ in the original sample or in the independent validation sample (IVS) (all *p* > 0.05).

**Table 1:** Demographic and clinical characteristics at baseline for SCZ participants in the original sample and in the independent validation sample, separated by their GAF score median split at the post-training timepoint.

	Original sample (training time = 40 h)				Independent validation sample (IVS) (training time = 50 h)				ORIG vs. IVS for GAF < 45		IVS vs. ORIG for GAF ≥ 45	
	GAF < 45 (*N* = 18)	GAF ≥ 45 (*N* = 18)	*t*/*χ*^2^	*p*	GAF < 45 (*N* = 10)	GAF ≥ 45 (*N* = 10)	*t*/*χ*^2^	*p*	*t*/*χ*^2^	*p*	*t*/*χ*^2^	*p*
Age	47 (9.10)	47 (9.0)	0.05	0.95	38 (13.2)	41 (11.84)	–0.45	0.65	–2.08	0.05	–1.52	0.14
Sex^a^	Male = 14	Male = 14	0.00	1.00	Male = 8	Male = 7	0.60	0.44	9.80	0.02	5.08	0.02
YoE	13 (1.7)	13 (1.9)	0.28	0.78	12.8 (2.0)	13.7 (3.1)	–0.73	0.47	–0.81	0.42	0.46	0.65
CPZ^b^	265 (129.4)	299 (212.5)	–0.50	0.62	399 (354.9)	290 (188.2)	0.82	0.42	1.26	0.22	0.45	0.64
Illness duration	26 (15.4)	27 (10.1)	0.15	0.87	20.4 (14.4)	19.3 (11.1)	0.18	0.86	–0.90	0.34	0.98	0.33
*Clinical baseline*												
PANSS positive	16.6 (5.3)	15.3 (5.8)	0.69	0.49	21.1 (6.1)	19.6 (6.4)	0.54	0.59	2.12	0.04	1.74	0.09
PANSS negative	16.7 (5.1)	15.9 (6.1)	0.38	0.70	21.9 (6.4)	16.1 (6.1)	2.06	0.05	2.44	0.02	0.06	0.95
PANSS general	35 (7.5)	30.6 (9.1)	1.57	0.12	41.8 (8.3)	36.0 (4.2)	1.24	0.23	2.28	0.03	1.27	0.21
PANSS total	68.2 (13.6)	61.8 (17.2)	1.23	0.22	84.8 (16.4)	71.7 (22.9)	1.49	0.15	2.94	0.00	1.27	0.21
GAF	42.9 (6.2)	48.8 (8.8)	–2.29	0.02	43.4 (10.7)	49.6 (12.3)	–1.10	0.28	0.14	0.88	0.18	0.85
*Clinical post training*												
PANSS positive	17.3 (4.5)	13.5 (3.3)	3.70	0.00	19.4 (4.3)	17.8 (5.6)	0.71	4.82	1.19	0.24	3.12	0.00
PANSS negative	16.0 (4.7)	14.1 (5.6)	1.12	0.26	23.4 (5.5)	15.0 (7.5)	2.88	0.01	3.83	0.00	0.37	0.71
PANSS general	34.6 (7.9)	28.1 (7.5)	2.53	0.02	43.4 (10.9)	37.9 (11.9)	1.07	0.30	2.49	0.01	2.61	0.00
PANSS total	67.9 (13.4)	54.9 (13.9)	2.92	0.00	86.1 (14.6)	70.7 (23.6)	1.79	0.09	3.41	0.00	2.23	0.03
GAF	40.2 (3.1)	54.6 (8.07)	–7.06	0.00	37.4 (7.3)	56.6 (7.4)	–5.82	0.00	–1.47	0.15	1.71	0.09

*YoE* years of education, *CPZ* chlorpromazine equivalent, *GAF* Global Assessment of Functioning, *PANSS* Positive and Negative Syndrome Scale.

### Behavioral analysis

Repeated measures analysis of variance (ANOVA) revealed a main effect of time in almost all cognitive domains, including: processing speed, attention, verbal learning, visual learning, and global cognition (see Table 2). We found a significant interaction of time and group in working memory (*F* = 8.4, *p* = 0.006) and verbal learning (*F* = 4.34, *p* = 0.04) domains that are the main treatment targets of the auditory CT.

**Table 2:** Cognitive measures at baseline and at the post-training timepoint in SCZ patients with poor and good functional outcome.

	Poor (*N* = 21)		Good (*N* = 19)		Main effect of time *F* (*p*)	Interaction (Group × Time) *F* (*p*)	Cohen’s *d*
	Baseline (SD)	Post-training (SD)	Baseline (SD)	Post-training (SD)			
Global cognition	32.1 (14.0)	34.4 (14.5)	30.1 (16.6)	35.1 (13.4)	11.0 (0.002)*	1.6 (0.2)	–0.18
Attention	40.2 (15.8)	43.8 (12.8)	39.9 (14.0)	42.7 (12.6)	4.1 (0.05)*	0.0 (0.8)	0.05
Speed of processing	36.2 (14.0)	38.6 (17.8)	37.8 (15.4)	40.9 (14.4)	5.0 (0.03)*	0.008 (0.7)	–0.04
Executive function	41.3 (11.8)	41.2 (11.3)	41.6 (13.4)	41.3 (9.6)	0.02 (0.86)	0.004 (0.94)	0.02
Working memory	44.5 (12.2)	40.9 (11.1)	36.8 (15.0)	40.3 (12.4)	0.0 (0.961)	8.6 (0.006)*	–0.51
Verbal memory	31.6 (13.8)	31.4 (17.0)	30.5 (16.0)	30.8 (14.3)	0.003 (0.98)	0.53 (0.90)	–0.03
Verbal learning	37.6 (7.1)	38.1 (9.0)	35.2 (9.3)	40.1 (8.7)	6.94 (0.01)*	4.34 (0.04)*	–0.52
Visual learning	37.6 (14.4)	42.2 (12.3)	36.5 (15.5)	41.9 (14.4)	7.2 (0.01)*	0.04 (0.8)	–0.07

*Level of significance *p* < 0.05.

### Performance of original classification model

We found that SCZ subjects with GAF ≥ 45 after ABCT showed significant improvement in GAF scores from baseline (*t* = 2.2, *p* = 0.05). Overall, the sMRI GM classifier correctly discriminated SCZ patients in the original sample with higher functioning from lower functioning responsiveness to the CT intervention with a cross-validated balanced accuracy (BAC) of 69.4%, sensitivity = 72.2%, specificity = 66.7%, negative predictive value (NPV) = 70.6%, and number needed to diagnose (NND) of 2.6. Based on averaging 50 best test performance models (CV2), the 95% CI of our model resulted in 67.2 ± 4.43. The permutation analysis showed that the classification models produced by the binary GAF classifier in response to ABCT were highly significant at *p* < 0.001.

**Fig. 1:**
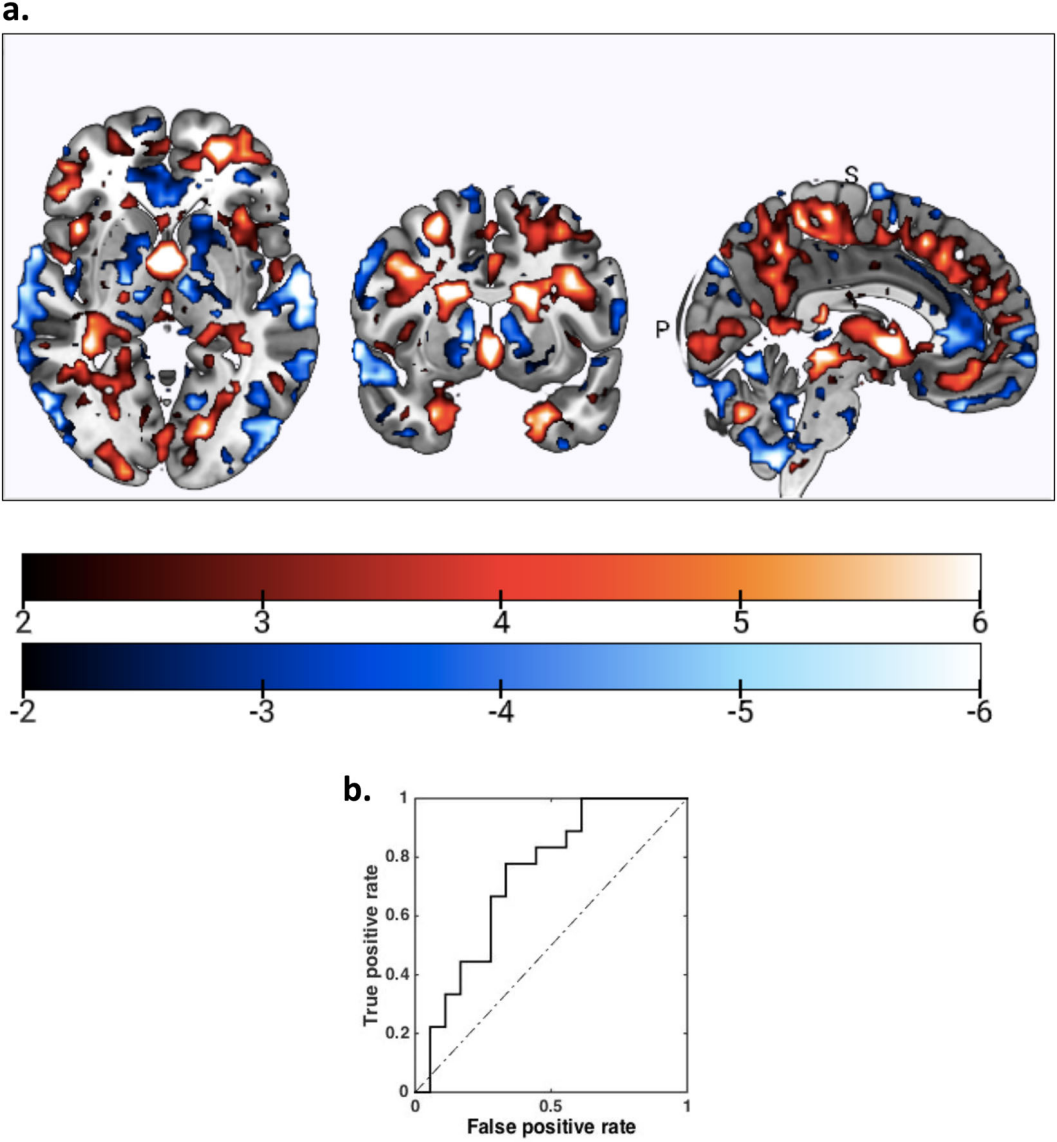
Structural MRI-based classifiers predict functional response to auditory-based cognitive training. **a**. The reliability of predictive pattern elements in significant outcome classification models was measured in terms of a cross-validation ratio (CVR) map (CVR = mean(w) /standard error(w), where w is the normalized weight vectors of the SVM models. Warm color scales indicate greater vs. lower GM volumes in the SCZ subsample with post-training GAF < 45 vs. GAF ≥ 45. Cool colors indicate greater vs. lower GM volumes in the GAF ≥ 45 vs. GAF < 45 subsamples. **b**. Receiver-operator-curve of the class probability values obtained from the trained model in unseen SCZ persons, as determined by nested cross-validation.

Inspection of the mean feature weights generated within the CV framework revealed that the classification of the higher functioning from lower functioning patients in response to the ABCT intervention was driven by greater baseline GM volumes in primarily temporal regions (i.e., in bilateral superior and inferior temporal regions, including ventral visual word form area, and parahippocampal gyri), thalamic and frontal regions in the ACC, as well as greater GM volumes in the posterior cingulate cortex and cerebellum (Fig. 1). Though the GM pattern was mainly characterized by higher baseline volumes in the higher functioning patients, the lower functioning group also showed higher volumes in primarily motor (i.e., premotor cortex and supplementary motor area) and caudate regions within the basal ganglia (Fig. 1).

### Out of cross-validation (OOCV) model performance

We next applied the original GM classification model to the IVS to predict follow-up functioning after the ABCT intervention in order to test whether the original GM classification model would generalize to the IVS. The model was able to successfully discriminate lower from higher functioning SCZ patients in the IVS at post ABCT sufficiently above chance with a BAC of 62.5%, sensitivity 90.9% and specificity 33.3%, NPV of 75.0 and NND of 4.1.

Although the MRI classification model provided accurate estimates (e.g., BAC = 69.4%) of correctly discriminating higher vs. lower functioning in response to the ABCT, we also wanted to ensure that the MRI-based classifiers did not predict generic baseline variations in global functioning that were not specific to the ABCT intervention. To investigate this possibility further, we replaced the GAF post ABCT functioning labels of the SCZ patients at follow-up with the respective classification labels derived from the baseline GAF scores and repeated the SVM analyses with the same machine learning pipeline as described previously. The MRI classifier differentiated lower from higher functioning SCZ with a non-significant classification at chance level with a BAC 44.4%. These convergent results indicate the robustness and reliability of our results and reveal that despite the use of different scanners and different samples of patients in the original sample and the IVS, we found that individual structural GM features strongly predicted a specific therapeutic response to the ABCT intervention in both the original and IVS samples, and were no due to general baseline differences in functioning levels.

### Decision scores and correlational analysis

The decision values of the discriminative GM signature for lower vs. higher functioning after ABCT were not associated with the baseline functioning levels (all *p* > 0.05), indicating that the prediction of response to ABCT was not confounded by baseline patterns but was specific to predicting response to the intervention.

We investigated the relationship between decision scores in both the original and OOCV models with Positive and Negative Syndrome Scale (PANSS) symptoms and illness duration as well as decision scores with sex, in order to exclude the possibility that the original classification was biased by respective differences within and between the two samples. None of the associations yielded significant results (all *p* > 0.05).

We also correlated decision scores with antipsychotic medication dosage as assessed via CPZ equivalents, and found significant associations (*r* = 0.42, *p* < 0.02) in the original sample, suggesting that higher doses of medication were associated with better functioning after the ABCT intervention. No such correlation between decision scores and CPZ medication was observed in the IVS (*r* = 0.08, *p* = 0.53).

## Discussion

This study applied MRI-based machine learning to predict individual functional responses to an intensive course of ABCT in chronically-ill SCZ patients. The original classification model provided accurate estimates of 69.4% in correctly discriminating higher vs. lower functioning in response to the ABCT intervention. Importantly, the MRI-based classifiers did not predict baseline variations in functioning, indicating that the individual structural features were therapeutically specific to predicting response to the ABCT intervention. The original classification model generalized to an IVS with an accuracy of 62.5%. These results confirm that GM volumes have high predictive specificity for individual therapeutic functional response to our ABCT intervention in SCZ patients, and demonstrate that MVPA methods provide great potential to use neuroanatomical biomarkers to predict functional response to therapeutic interventions at the individual level^24,25^.

We used a median split of GAF score of 45, which significantly differentiated the SCZ who showed higher versus lower functioning in response to the CT intervention. Two prior studies have shown that, at the group level, SCZ patients with higher GM volumes at baseline showed a stronger response to CT^1,12^. GM volumes have also been shown to increase in response to CT interventions^13^. However, group-level analyses cannot take into account the substantial individual neuroanatomical heterogeneity that occurs at the individual level in SCZ^26^. The goal of precision-medicine is to select and adapt therapeutic approaches based on each patient’s individual neural and clinical characteristics^27,28^. In order to take into account individual neuroanatomical heterogeneity at baseline and reliably validate the origin of the predictive information, we replaced patients’ GAF scores at post ABCT with their baseline scores and repeated the sMRI GM analysis. Strikingly, we were not able to find significant GM patterns that successfully discriminated patients with lower from higher functioning at baseline. Moreover, the decision values of the discriminative GM signature for lower vs. higher functioning were not associated with baseline functioning levels. These convergent results together indicate that the neuroanatomical classifier accurately predicted functional response that was specific to the ABCT therapeutic intervention, and was not due to general non-specific differences in baseline GM patterns or functioning.

Accurate discrimination of participants with higher functioning after the CT intervention (GAF scores of ≥45) was specifically shown by greater baseline GM volume pattern in superior temporal gyrus (STG), ventral visual form areas, thalamus, and parahippocampal gyri. Longitudinal studies indicate progressive decreases in STG volume after the first psychotic episode, and this neuroanatomical abnormality is consistently reported in people with established SCZ^29,30^. These results are consistent with our prior group-based studies showing the functional importance of the STG during ABCT, and its responsiveness to our ABCT interventions^18,22^. We have previously shown increased recruitment of the primary auditory cortex and the prefrontal cortex mediating improved auditory learning after ABCT^22^. In addition, the results from our recent study indicate that at baseline, even chronically-ill SCZ suffering from hallucinations were able to recruit the STG, extending into ventral occipital temporal regions within the visual ventral word form area, which correlated with auditory and verbal working memory^31^. Our data suggest that an intact GM reserve particularly in the STG at baseline drives plasticity in response to auditory CT interventions, and is likely to predict which SCZ patients will receive most benefit from auditory training interventions.

We have previously shown at a group level that the intact structure of thalamic-prefrontal regions was also an important determinant of successful responsivity to CT interventions in SCZ^14^. In addition, Ramsay et al. demonstrated the mechanisms of how improved recruitment of thalamic-prefrontal activity and connectivity after CT predicted overall improvements in cognitive functions^32^. Importantly, previous studies have also consistently shown that the ACC/mPFC plays a critical role in supporting higher-order cognitive control functions that are important for conflict resolution and reality-monitoring functions^33–35^. For example, we have previously delineated how increased recruitment of the ACC/mPFC induced by our CT regimen predicted successful reality-monitoring performance that generalized to improvements in long-term social functioning at a group level^9^. These prior studies support the data in the present study, in which we found that greater GM volume in the thalamic-ACC regions at baseline predicted better functioning after our CT regimen.

Interestingly, we also found that SCZ who showed greater GM volume in the cerebellum at baseline also revealed better overall functioning induced by ABCT. The cerebellum is important for mediating sensory prediction-errors for updating an internal model of implicit learning and action-outcome behaviors that are fundamental for improving real-world functioning in SCZ^36–38^. These data are consistent with the neuroplasticity principles of our ABCT intervention, which specifically trains SCZ to improve auditory detection, temporal integration, prediction-error and learning, which have shown to directly contribute to higher-level functioning^18,39^.

The low and the high functioning groups also differed with respect to their cognitive response to ABCT. In particular, the neuroimaging pattern showing patients with higher baseline GM reserve in STG that is critical for verbal learning is consistent with the cognitive improvements in verbal learning observed in the high functioning group induced by the ABCT intervention. Similarly, patients who had greater thalamic-prefrontal and cerebellum baseline reserve in GM pattern that are critical for updating prediction-error learning for working memory^9,11,32,39^ also showed improved working memory observed in the high functioning group that was induced by the ABCT intervention. These results reveal the functional importance of greater GM preserve in particular within STG, thalamic-PFC, and cerebellar regions at baseline that predict responsiveness to our ABCT intervention.

In summary, SCZ patients who exhibit a pattern of greater GM structural volumes at baseline in STG, thalamus, ACC, and cerebellum, in particular, may possess the needed neurological infrastructure to maximally benefit functionally from intensive ABCT. Together, our present findings are consistent with these prior meta-analyses and group-based studies, indicating that the individual predictive value of recruitment of regions particularly in the cerebellum, STG, and thalamic-prefrontal areas is important and critical targets for CT interventions^4^.

It must also be noted that we found that accurate discrimination of SCZ participants with lower functioning (i.e., GAF score of <45) was characterized by greater GM volumes in premotor and basal ganglia regions. Aberrant connectivity in the motor system and disturbances in motor behavior have been observed in SCZ patients with lower functioning^29,30^. Higher basal ganglia volumes (hypothesized to be due to striatal hyperdopaminergia) have also been shown in both medicated and antipsychotic-naive patients in meta-analytic studies^37^ concurrent with motor disturbances as one of the central clinical features of SCZ.

Some studies have raised the question as to whether GM preservation or loss can be attributed to cumulative exposure to antipsychotic medications, rather than to aberrant neural developmental processes^40^. Importantly, the decision values of our SVM analysis that accurately predicted higher functioning in response to the CT intervention showed a significant relationship with medication dosage. Specifically, SCZ patients who had a higher medication dosage at baseline as well as lower positive symptoms also had better functioning following the intervention. Taken together, these findings suggest that structural features together with medication dosage provide useful determinants of individual functional responsiveness to CT interventions at the single-subject level.

The present findings provide the founding basis for the prediction of functional response to ABCT interventions that may be reliably enhanced using neuroanatomical pattern recognition at a single baseline timepoint operating at the single-subject level. Here, rather than investigating MRI GM volume change at multiple time-points from pre-to-post intervention to predict functional outcome change, we use MRI-based biomarkers solely at the single baseline timepoint to test if we are able to identify predictive biomarkers in individuals who show greatest functional response to ABCT interventions. The limitation of the present study is that the findings here do not account for the heterogeneity associated with additional neurophysiologic, environmental, and genetic factors at baseline that may play a role in the response to ABCT. In order to develop a more robust and definitive baseline predictive model, future studies will require: (1) a wide variety of behavioral and neurophysiologic data analyzed in a multivariate fashion to develop more accurate predictive baseline biomarkers, so that meaningful signals are less likely to be lost due to noise from highly variable and heterogeneous metrics; (2) larger study samples from a wide variety of multisite studies, in order to provide more extensive geographical generalizability; and (3) participants with a range of illness durations. The patients in our study had been ill for more than 20 years, limiting the generalizability of our findings to only older people with chronic illness (4) implementation of multimodal imaging data (e.g., that combines structural and functional neuroimaging data) for neuromonitoring of early response to interventions^41–43^.

Using whole-brain MVPA analyses, we have identified a structural MRI fingerprint associated with preserved GM volumes within particular regions in the STG, thalamus, ACC, and cerebellum that predicted improved functioning following an ABCT intervention, and that serves as a model for how to facilitate precision clinical therapies for SCZ based on imaging data. Future studies should investigate if the individuals with greatest GM loss in these regions (who may also have the greatest vulnerability for more subsequent and severe psychotic episodes) can benefit from more intensive and integrative therapies of combined pharmacotherapy, CT^44^ and neuromodulation^45^. If identified early in young adulthood, cerebellum-temporal-thalamic-prefrontal GM loss may reflect an important indicator to provide early and intensive interventions to mitigate and reduce the impact of future and more severe psychotic episodes on functioning^46,47^.

## Methods

### Participants and procedure

Two independent samples of SCZ participants who had structural imaging data were drawn from two larger clinical trials (ClinicalTrials.gov Identifier: NCT02105779)^48,49^ and (ClinicalTrials.gov NCT00312962)^11^. As is customary in predictive analytics, the MVPA model was constructed from the first set of subjects (the original sample, *N* = 44) and then applied to a different set of subjects (the independent sample, *N* = 23) using an OOCV approach. This process produces an unbiased estimate of the method’s predictive accuracy on new individuals rather than merely fitting the current study population^50^. The study was carried out in accordance with The Declaration of Helsinki, and reviewed by the Institutional Review Board at the University of California, San Francisco. All participants provided written consent.

**Fig. 2:**
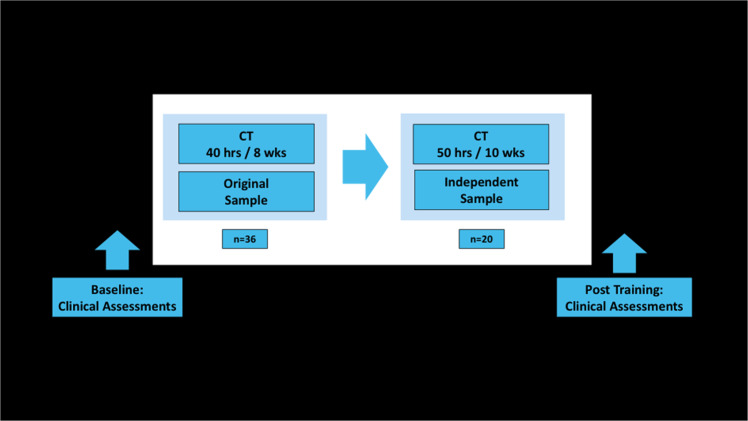
Training design of the original and independent validation samples. The machine learning support vector model reliably predicted GAF ≥ 45 vs. GAF < 45 in SCZ participants in response to auditory-based cognitive training (CT) at the single-subject level in both samples.

All SCZ subjects were recruited from community mental health centers and outpatient clinics. Inclusion criteria were: Axis I diagnosis of SCZ, schizoaffective disorder, or psychosis not otherwise specified (determined by the Structured Clinical Interview for DSM-IV [SCID])^51^. All participants provided written informed consent and then completed structural imaging and assessments measuring clinical and real-world functioning at baseline and after the CT intervention. Participants with poor signal-to-noise ratio in their neuroanatomical images were excluded from the final analyses for the original (*N* = 5) and IVS (*N* = 1). In addition, three participants from the original sample and two participants from the IVS cohort did not complete functioning assessments at the follow-up timepoint. An overview of our procedures can be found in Fig. 2.

### Auditory-based CT intervention

The design of our neuroscience-informed computerized CT intervention is based on 3 decades of research into known mechanisms of neural plasticity, which have been shown to increase neuronal activity, synaptic connectivity, and neuronal fiber integrity^52,53^. In particular, research has documented the neural plasticity of cortical responses as an individual acquires new perceptual and cognitive abilities^54^. A rich body of work shows that improved functioning after our CT regimens specifically results from neuroplasticity (defined as changes in neural structure and real-world functioning that are induced by the CT intervention)^9,10,28,54^. Complete details of the ABCT exercises can be found at http://www.positscience.com/our-products/brain-fitness-program. In the original sample, each SCZ patient completed the same amount of auditory training exercises for 1 h a day for a total of 40 h of training. Similarly, each SCZ patient in the IVS sample completed the same amount of training, performed for 1 h a day for a total of 50 h over the course of 10 weeks. In the exercises, patients were driven to make progressively more accurate discriminations and temporal integration about the spectro-temporal fine-structure of auditory stimuli under conditions of increasing working memory load under progressively briefer presentations, and to incorporate and generalize those improvements into working memory rehearsal and decision-making. The auditory exercises were continuously adaptive: they first established the precise parameters within each stimulus set required for an individual subject to maintain 80% correct performance, and once that threshold was determined, task difficulty increased systematically and parametrically as performance improved^20^.

### Clinical and functional outcome assessments

The Structured Clinical Interview (SCID for DSM-IV) Axis I Disorders^51^ was administered at baseline to all participants. In both the original GM machine learning and the IVS samples, the GAF Scale of the DSM-IV^55^ was used to assess functioning at baseline and after the CT intervention, and the PANSS^56^ was used to assess severity of clinical symptoms at baseline and after the CT intervention. Functional assessments such as the GAF quantify measures of real-world day-to-day functioning on a scale of 0–100. Cognitive performance was assessed with the MATRICS Consensus Cognitive Battery^57^. All cognitive outcome measures were distinct and independent from tasks practiced during training. Clinical researchers who conducted diagnostic and clinical interviews (SCID, PANSS, GAF) first completed extensive training on testing, interviewing, and scoring criteria of individual items (e.g., including scored videotaped sessions; observed sessions conducted by experienced psychiatrists and psychologist staff; as well as several mock practice sessions). GAF ratings were made at the conclusion of the interview using information obtained during the symptom and functioning assessments, yielding excellent intraclass correlation coefficients that were greater than 0.85^11^, in line with prior studies^58^. All clinical researchers who were trained on diagnostic and clinical interviews and scoring were blind to the condition that each patient was assigned.

### Machine learning strategy

Following our aims, we employed a nested cross-validated machine learning pipeline to evaluate the sensitivity of GM volumetric features at baseline to predict GAF functional response to CT at a single-subject level, using a median split strategy as validated in our prior studies with the machine learning analyses pipeline delineated below^24,25^. GAF scores were used to determine labels of lower vs. higher functioning in response to CT by setting a median split as a cutoff. Taking our chronic SCZ patient populations into account (i.e., defined as patients with ~20 years of illness duration), a GAF score of 45 indicated the most representative level of functioning that the chronic SCZ patient population experienced (e.g., the range of GAF scores in the chronic SCZ patients was from 29 to 67 in the original sample and 23 to 70 in the OOCV sample at baseline). Thus, a GAF ≥ 45 determined the selection criteria for patients with higher functioning (*n* = 18) whereas GAF < 45 determined the selection criteria for patients with lower functioning (*n* = 18). In our OOCV analysis, the median split cutoff was identical, with 10 SCZ with lower and 10 SCZ with higher functioning in the IVS. Demographic characteristics of the two samples, separated by their GAF score median split at the post-training timepoint, are presented in Table 1. GAF score distributions can be found in Supplementary materials (Supplementary Figs. 1 and 2).

### Neuroimaging protocol

In the original sample, imaging was performed on a 3 Tesla Siemens Prisma MRI scanner with 64- and 20-channel head and neck coils at the Neuroscience Imaging Center at University of California San Francisco. In the IVS, high-resolution anatomical images were acquired from each individual on a 3T General Electric Signa LX 15 scanner, utilizing 3D magnetization prepared rapid gradient echo MRI. Imaging parameters were: 160 1-mm slices; FOV = 256 mm, matrix = 256 × 256, TE = 2 ms, TR = 7 ms, flip = 15. The manual of the CAT12 toolbox, version r > 1200 (http://www.neuro.uni-jena.de/cat12/CAT12-Manual.pdf) details the preprocessing steps applied to the structural images^25^.

### MRI processing pipeline

The manual of the CAT12 toolbox, version r>1200 (http://www.neuro.uni-jena.de/cat12/CAT12-Manual.pdf) details the processing steps applied to the structural images^25^. These steps consist of:

1. A 1st denoising step based on Spatially Adaptive Non-Local Means filtering^59^.
2. An Adaptive Maximum A Posteriori (AMAP) segmentation technique, which models local variations of intensity distributions as slowly varying spatial functions and thus achieves a homogeneous segmentation across cortical and subcortical structures.
3. A 2nd denoising step using Markov Random Field approach that incorporates spatial prior information of adjacent voxels into the segmentation estimation generated by AMAP^60^.
4. A Local Adaptive Segmentation (LAS) step, which adjusts the images for white matter (WM) inhomogeneities and varying GM intensities caused by differing iron content in, e.g., cortical and subcortical structures. The LAS step is carried out before the final AMAP segmentation.
5. A Partial Volume Segmentation algorithm that is capable of modeling tissues with intensities between GM and WM, as well as GM and cerebrospinal fluid and is applied to the AMAP-generated tissue segments.
6. A high-dimensional DARTEL registration of the image to a MNI-template generated from the MRI data of 555 healthy controls in the IXI database (http://www.braindevelopment.org). The registered GM images were multiplied with the Jacobian determinants obtained during registration to produce GM volume maps.
7. GM images were smoother with a Gaussian smoothing kernel with a 4 mm full width at half maximum.

The Quality Assurance framework of CAT12 was used to empirically check the quality of the GMV maps. By computing the correlation of each image to all other images, taking the original and independent sample separately, we removed two images whose correlation exceeded 2 standard deviations from the sample mean due to MRI artifacts.

### Machine learning preprocessing parameters

We regressed out the effect of the total GM volumes by entering the values as a covariate. In the inner CV loop, zero-variance features were pruned. Then, a dimensionality reduction procedure was applied through principal component analysis (PCA) in order to minimize the generalization error. PCA was applied to 62,188 voxels contained in GM volume images used in the analysis. PCA projected the image information to a limited number of 80 eigenvariates (80 PCs) in the CV1 training data that were subsequently scaled (0–1) and then forwarded to the SVM linear machine learning algorithm described here: the optimal hyperparameter *C* was determined using grid search defined by 11 parameters in the range *C* = [0.0156–16].

### Machine learning analysis pipeline

The in-house machine learning platform NeuroMiner, version 1.0^25^ was used to set up a machine learning analysis pipeline for the individual classification ability (higher SCZ functioning vs. lower SCZ functioning) based on GM volumes for the prediction GAF target. To strictly separate the training process from the evaluation of the predictor’s generalization capacity and prevent the leakage of information and overfitting, the pipeline was completely embedded into a nested cross-validation (CV) framework^61^ with a 10-by-5 CV structure for both inner (CV1) and outer (CV2) cycles. All steps of SVM training, including feature selection and parameter optimization, were performed on the CV1 training and validation partitions, while the generalization error was exclusively estimated from the CV2 test samples. Importantly, the SVM training does not reoccur on the outer cycle (CV2). This procedure was applied to each fold for each permutation combination independently. This analysis chain was applied to the outer CV cycle employing a linear SVM algorithm that learns to separate hyperplane in the concatenated PC kernel space that maximizes the geometric margin between the most similar instances (=support vectors) of the low and high functioning groups. We determined the patients’ membership to higher vs. lower functioning using majority voting. The majority voting ensemble combines the predictions from multiple CV2 folds to decide which class (high or low functioning) should be considered as the winning predictive class for achieving better performance. Model’s performance was measured by BAC, sensitivity, specificity, positive predictive value, NPV, and NND. Statistical significance of the final prediction set was assessed through permutation testing, with *α* = 0.05 and 1000 permutations. Further information on this approach can found in Koutsouleris et al.^61^.

### Statistical analysis

Sociodemographic differences between groups were examined using ANOVA for parametric data, and by *χ*^2^ test for non-parametric data, as implemented in Jamovi for Windows (0.9.5.12). We conducted repeated measures ANOVA with time as the within-subject factor (baseline, follow-up) and group as the between subject factor (high vs. low functioning) on cognitive outcomes. Furthermore, potential interactions between subjects SVM decision scores and their (1) GAF levels, (2) antipsychotic medication doses (in chlorpromazine equivalents), and (3) clinical symptoms at baseline and follow-up were assessed by means of correlational analysis (Pearson’s *r*). Significance was defined at *p* < 0.05.

### Reporting summary

Further information on research design is available in the Nature Research Reporting Summary linked to this article.

## Supplementary information

Reporting Summary

Supplementary Information

## Data Availability

The data that support the findings of this study are available on request from the corresponding author K.S. The data are not publicly available due to ethical restrictions protecting patients’ privacy and consent.

## Code availability

The Neurominer Software code is available for download at https://github.com/neurominer-git.
