# Author Correction: Multivariate pattern analysis of brain structure predicts functional outcome after auditory-based cognitive training interventions

**DOI:** 10.1038/s41537-021-00177-w

**Published:** 2021-09-27

**Authors:** Lana Kambeitz-Ilankovic, Sophia Vinogradov, Julian Wenzel, Melissa Fisher, Shalaila S. Haas, Linda Betz, Nora Penzel, Srikantan Nagarajan, Nikolaos Koutsouleris, Karuna Subramaniam

**Affiliations:** 1 Faculty of Medicine and University Hospital of Cologne, University of Cologne, Cologne, Germany; 2 Department of Psychiatry and Psychotherapy, Ludwig-Maximilian-University, Munich, Germany; 3 Department of Psychiatry, University of Minnesota, Minneapolis, MN USA; 4 Department of Psychiatry, Icahn School of Medicine at Mount Sinai, New York, NY USA; 5 Department of Basic Medical Sciences, Neuroscience and Sense Organs—University of Bari Aldo Moro, Bari, Italy; 6 Department of Radiology and Biomedical Imaging, University of California San Francisco, San Francisco, CA USA; 7 Institute of Psychiatry, Psychology and Neuroscience, King’s College London, London, UK; 8 Max Planck Institute of Psychiatry, Munich, Germany; 9 Department of Psychiatry, University of California San Francisco, San Francisco, CA USA

**Keywords:** Schizophrenia, Neuroscience

Correction to: *npj Schizophrenia* 10.1038/s41537-021-00165-0, published online 19 August 2021

In the original version of this Article, references 59 and 60 were swapped. This has now been corrected in both the HTML and PDF version of the Article.

