# Supplementary material for: Multivariate pattern analysis of brain structure predicts functional outcome after auditory-based cognitive training interventions
Source: NPJ Schizophr. 2021 Aug 19;7:40. doi: 10.1038/s41537-021-00165-0 (PMC8376975; doi:10.1038/s41537-021-00165-0)
Supplement: Supplementary file 1 — Reporting Summary [file 41537_2021_165_MOESM1_ESM.pdf]

nature research

Corresponding author(s): DBPR NPJSCHZ-00624

Last updated by author(s): May 14, 2021

# Reporting Summary

Nature Research wishes to improve the reproducibility of the work that we publish. This form provides structure for consistency and transparency in reporting. For further information on Nature Research policies, see our Editorial Policies and the Editorial Policy Checklist.

## Statistics

For all statistical analyses, confirm that the following items are present in the figure legend, table legend, main text, or Methods section.

| n/a | Confirmed | |
|---|---|---|
| ☐ | ☒ | The exact sample size (*n*) for each experimental group/condition, given as a discrete number and unit of measurement |
| ☐ | ☒ | A statement on whether measurements were taken from distinct samples or whether the same sample was measured repeatedly |
| ☐ | ☒ | The statistical test(s) used AND whether they are one- or two-sided<br>*Only common tests should be described solely by name; describe more complex techniques in the Methods section.* |
| ☐ | ☒ | A description of all covariates tested |
| ☐ | ☒ | A description of any assumptions or corrections, such as tests of normality and adjustment for multiple comparisons |
| ☐ | ☒ | A full description of the statistical parameters including central tendency (e.g. means) or other basic estimates (e.g. regression coefficient) AND variation (e.g. standard deviation) or associated estimates of uncertainty (e.g. confidence intervals) |
| ☐ | ☒ | For null hypothesis testing, the test statistic (e.g. *F*, *t*, *r*) with confidence intervals, effect sizes, degrees of freedom and *P* value noted<br>*Give P values as exact values whenever suitable.* |
| ☒ | ☐ | For Bayesian analysis, information on the choice of priors and Markov chain Monte Carlo settings |
| ☒ | ☐ | For hierarchical and complex designs, identification of the appropriate level for tests and full reporting of outcomes |
| ☐ | ☒ | Estimates of effect sizes (e.g. Cohen's *d*, Pearson's *r*), indicating how they were calculated |

*Our web collection on statistics for biologists contains articles on many of the points above.*

## Software and code

Policy information about availability of computer code

| | |
|---|---|
| Data collection | No software was used for data collection apart from Brain HQ Posit Science software that was used to cognitively train the participants. |
| Data analysis | Neurominer software available on github was used for the data analysis and it is available online https://github.com/neurominer-git/NeuroMiner-1 |

For manuscripts utilizing custom algorithms or software that are central to the research but not yet described in published literature, software must be made available to editors and reviewers. We strongly encourage code deposition in a community repository (e.g. GitHub). See the Nature Research guidelines for submitting code & software for further information.

## Data

Policy information about availability of data

All manuscripts must include a data availability statement. This statement should provide the following information, where applicable:

- Accession codes, unique identifiers, or web links for publicly available datasets
- A list of figures that have associated raw data
- A description of any restrictions on data availability

The data that support the findings of this study are available on request from the corresponding author K.S. The data are not publicly available due to ethical restrictions; if not applied sharing could compromise participants' privacy.

# Field-specific reporting

Please select the one below that is the best fit for your research. If you are not sure, read the appropriate sections before making your selection.

☐ Life sciences ☒ Behavioural & social sciences ☐ Ecological, evolutionary & environmental sciences

For a reference copy of the document with all sections, see nature.com/documents/nr-reporting-summary-flat.pdf

# Behavioural & social sciences study design

All studies must disclose on these points even when the disclosure is negative.

| | |
|---|---|
| Study description | This study represents the imaging component of two larger double-blind randomized clinical trials of cognitive training in schizophrenia in with quantitative experimental data |
| Research sample | Two independent samples of schizophrenia participants who had structural imaging data were drawn from two larger clinical trials (ClinicalTrials.gov Identifier: NCT02105779) and (ClinicalTrials.gov NCT00312962). |
| Sampling strategy | This is randomized clinical trial of cognitive training in schizophrenia. With power calculations based on our previous cognitive training studies, assuming 20% attrition, we would need a minimum of 36 subjects in the original sample at all baseline and post-training time-points to attain medium-large effect sizes of Cohen's d of at least 0.5 to detect significant change in neural, cognitive and functional outcome between groups in univariate analysis. |
| Data collection | All SCZ subjects were recruited from community mental health centers and outpatient clinics. Inclusion criteria were: Axis I diagnosis of schizophrenia, schizoaffective disorder, or psychosis not otherwise specified (NOS) (determined by the Structured Clinical Interview for DSM-IV [SCID]). Data were collected by pen-pencil methods while tablets were used only for the training of the study participants. No one was present besides the patient and the research during the assessement. Importantly, the researcher was blind to the condition of the patient. |
| Timing | Study start date was April 7, 2014 till April 5, 2019. |
| Data exclusions | The exclusion criteria were not entirely pre-established they were set due to assuring good data quality. Namely, participants with poor signal-to-noise ratio in their neuroanatomical images were excluded from the final analyses for the original (N=5) and independent validation samples (IVS) (N=1). Additionally, three participants from the original sample and two participants from the IVS cohort did not complete functioning assessments at the follow-up time point |
| Non-participation | In the present imaging study, within the original sample, three participants from the original sample and two participants dropped out from baseline to follow-up due to the intensive nature of the cognitive training and assessments. |
| Randomization | In the larger double-blind RCT, participants were matched on age, gender and education and then randomized into each group. At a group level at baseline, there was no difference in medication, symptoms or illness duration, as was the case within the imaging component of the present study. |

# Reporting for specific materials, systems and methods

We require information from authors about some types of materials, experimental systems and methods used in many studies. Here, indicate whether each material, system or method listed is relevant to your study. If you are not sure if a list item applies to your research, read the appropriate section before selecting a response.

## Materials & experimental systems

| n/a | Involved in the study |
|---|---|
| ☐ | ☐ Antibodies |
| ☐ | ☐ Eukaryotic cell lines |
| ☐ | ☐ Palaeontology and archaeology |
| ☐ | ☐ Animals and other organisms |
| ☐ | ☐ Human research participants |
| ☐ | ☒ Clinical data |
| ☐ | ☐ Dual use research of concern |

## Methods

| n/a | Involved in the study |
|---|---|
| ☐ | ☐ ChIP-seq |
| ☐ | ☐ Flow cytometry |
| ☐ | ☒ MRI-based neuroimaging |

## Antibodies

| | |
|---|---|
| Antibodies used | *Describe all antibodies used in the study; as applicable, provide supplier name, catalog number, clone name, and lot number.* |
| Validation | *Describe the validation of each primary antibody for the species and application, noting any validation statements on the manufacturer's website, relevant citations, antibody profiles in online databases, or data provided in the manuscript.* |

# Eukaryotic cell lines

Policy information about cell lines

| | |
|---|---|
| Cell line source(s) | State the source of each cell line used. |
| Authentication | Describe the authentication procedures for each cell line used OR declare that none of the cell lines used were authenticated. |
| Mycoplasma contamination | Confirm that all cell lines tested negative for mycoplasma contamination OR describe the results of the testing for mycoplasma contamination OR declare that the cell lines were not tested for mycoplasma contamination. |
| Commonly misidentified lines (See ICLAC register) | Name any commonly misidentified cell lines used in the study and provide a rationale for their use. |

# Palaeontology and Archaeology

| | |
|---|---|
| Specimen provenance | Provide provenance information for specimens and describe permits that were obtained for the work (including the name of the issuing authority, the date of issue, and any identifying information). |
| Specimen deposition | Indicate where the specimens have been deposited to permit free access by other researchers. |
| Dating methods | If new dates are provided, describe how they were obtained (e.g. collection, storage, sample pretreatment and measurement), where they were obtained (i.e. lab name), the calibration program and the protocol for quality assurance OR state that no new dates are provided. |

☐ Tick this box to confirm that the raw and calibrated dates are available in the paper or in Supplementary Information.

| | |
|---|---|
| Ethics oversight | Identify the organization(s) that approved or provided guidance on the study protocol, OR state that no ethical approval or guidance was required and explain why not. |

Note that full information on the approval of the study protocol must also be provided in the manuscript.

# Animals and other organisms

Policy information about studies involving animals; ARRIVE guidelines recommended for reporting animal research

| | |
|---|---|
| Laboratory animals | For laboratory animals, report species, strain, sex and age OR state that the study did not involve laboratory animals. |
| Wild animals | Provide details on animals observed in or captured in the field; report species, sex and age where possible. Describe how animals were caught and transported and what happened to captive animals after the study (if killed, explain why and describe method; if released, say where and when) OR state that the study did not involve wild animals. |
| Field-collected samples | For laboratory work with field-collected samples, describe all relevant parameters such as housing, maintenance, temperature, photoperiod and end-of-experiment protocol OR state that the study did not involve samples collected from the field. |
| Ethics oversight | Identify the organization(s) that approved or provided guidance on the study protocol, OR state that no ethical approval or guidance was required and explain why not. |

Note that full information on the approval of the study protocol must also be provided in the manuscript.

# Human research participants

Policy information about studies involving human research participants

| | |
|---|---|
| Population characteristics | See above |
| Recruitment | See above; behavioral and social sciences |
| Ethics oversight | The ethical board of the University of California San Francisco approved the study protocol. |

Note that full information on the approval of the study protocol must also be provided in the manuscript.

# Clinical data

Policy information about clinical studies

All manuscripts should comply with the ICMJE guidelines for publication of clinical research and a completed CONSORT checklist must be included with all submissions.

| | |
|---|---|
| Clinical trial registration | Two independent samples of SCZ participants who had structural imaging data were drawn from two larger clinical trials (ClinicalTrials.gov Identifier: NCT02105779) and (ClinicalTrials.gov NCT00312962) |
| Study protocol | The full trial study protocol can be found at Two independent samples of SCZ participants who had structural imaging data were drawn from two larger clinical trials (ClinicalTrials.gov Identifiers: NCT021057799 & NCT00312962 |

| | |
|---|---|
| Data collection | See above |
| Outcomes | Primary outcomes included: Neuroimaging, cognitive, symptom and functional outcomes |

# Dual use research of concern

Policy information about dual use research of concern

## Hazards

Could the accidental, deliberate or reckless misuse of agents or technologies generated in the work, or the application of information presented in the manuscript, pose a threat to:

| No | Yes | |
|---|---|---|
| ☒ | ☐ | Public health |
| ☒ | ☐ | National security |
| ☒ | ☐ | Crops and/or livestock |
| ☒ | ☐ | Ecosystems |
| ☒ | ☐ | Any other significant area |

## Experiments of concern

Does the work involve any of these experiments of concern:

| No | Yes | |
|---|---|---|
| ☒ | ☐ | Demonstrate how to render a vaccine ineffective |
| ☒ | ☐ | Confer resistance to therapeutically useful antibiotics or antiviral agents |
| ☒ | ☐ | Enhance the virulence of a pathogen or render a nonpathogen virulent |
| ☒ | ☐ | Increase transmissibility of a pathogen |
| ☒ | ☐ | Alter the host range of a pathogen |
| ☒ | ☐ | Enable evasion of diagnostic/detection modalities |
| ☒ | ☐ | Enable the weaponization of a biological agent or toxin |
| ☒ | ☐ | Any other potentially harmful combination of experiments and agents |

# ChIP-seq

## Data deposition

☐ Confirm that both raw and final processed data have been deposited in a public database such as GEO.

☐ Confirm that you have deposited or provided access to graph files (e.g. BED files) for the called peaks.

| | |
|---|---|
| Data access links<br>*May remain private before publication.* | *For "Initial submission" or "Revised version" documents, provide reviewer access links. For your "Final submission" document, provide a link to the deposited data.* |
| Files in database submission | *Provide a list of all files available in the database submission.* |
| Genome browser session<br>(e.g. UCSC) | *Provide a link to an anonymized genome browser session for "Initial submission" and "Revised version" documents only, to enable peer review. Write "no longer applicable" for "Final submission" documents.* |

## Methodology

| | |
|---|---|
| Replicates | *Describe the experimental replicates, specifying number, type and replicate agreement.* |
| Sequencing depth | *Describe the sequencing depth for each experiment, providing the total number of reads, uniquely mapped reads, length of reads and whether they were paired- or single-end.* |
| Antibodies | *Describe the antibodies used for the ChIP-seq experiments; as applicable, provide supplier name, catalog number, clone name, and lot number.* |
| Peak calling parameters | *Specify the command line program and parameters used for read mapping and peak calling, including the ChIP, control and index files used.* |
| Data quality | *Describe the methods used to ensure data quality in full detail, including how many peaks are at FDR 5% and above 5-fold enrichment.* |

Software | *Describe the software used to collect and analyze the ChIP-seq data. For custom code that has been deposited into a community repository, provide accession details.*

## Flow Cytometry

### Plots

Confirm that:

☐ The axis labels state the marker and fluorochrome used (e.g. CD4-FITC).

☐ The axis scales are clearly visible. Include numbers along axes only for bottom left plot of group (a 'group' is an analysis of identical markers).

☐ All plots are contour plots with outliers or pseudocolor plots.

☐ A numerical value for number of cells or percentage (with statistics) is provided.

### Methodology

| | |
|---|---|
| Sample preparation | *Describe the sample preparation, detailing the biological source of the cells and any tissue processing steps used.* |
| Instrument | *Identify the instrument used for data collection, specifying make and model number.* |
| Software | *Describe the software used to collect and analyze the flow cytometry data. For custom code that has been deposited into a community repository, provide accession details.* |
| Cell population abundance | *Describe the abundance of the relevant cell populations within post-sort fractions, providing details on the purity of the samples and how it was determined.* |
| Gating strategy | *Describe the gating strategy used for all relevant experiments, specifying the preliminary FSC/SSC gates of the starting cell population, indicating where boundaries between "positive" and "negative" staining cell populations are defined.* |

☐ Tick this box to confirm that a figure exemplifying the gating strategy is provided in the Supplementary Information.

## Magnetic resonance imaging

### Experimental design

| | |
|---|---|
| Design type | Neuroanatomical scan, T1 sequence |
| Design specifications | n.a. no blocks, no task, resting design, neuroanatomical sequence |
| Behavioral performance measures | n.a. no variables recorded no button press during the T1 sequence |

### Acquisition

| | |
|---|---|
| Imaging type(s) | Structural neuroimaging, voxel based morphometry |
| Field strength | 3T |
| Sequence & imaging parameters | In the original sample, imaging was performed on a 3 Tesla Siemens Prisma MRI scanner with 64- and 20-channel head and neck coils at the Neuroscience Imaging Center at University of California San Francisco. In the validatino sample, high-resolution anatomical images were acquired from each individual on a 3T General Electric Signa LX 15 scanner, utilizing 3D magnetization prepared rapid gradient echo MRI. Imaging parameters were: 160 1-mm slices; FOV =256mm, matrix=256×256, TE =2ms, TR=7ms, flip=15. The manual of the CAT12 toolbox, version r>1200 (http://www.neuro.uni-jena.de/cat12/CAT12-Manual.pdf) details the preprocessing steps applied to the structural images. |
| Area of acquisition | Whole brain scan |

Diffusion MRI ☐ Used ☒ Not used

### Preprocessing

Preprocessing software

The manual of the CAT12 toolbox, version r>1200 (http://www.neuro.uni-jena.de/cat12/CAT12-Manual.pdf) .
(1) A 1st denoising step based on Spatially Adaptive Non-Local Means (SANLM) filtering. Smoothing: (7) GM images were smoother with a Gaussian smoothing kernel with a 4mm full width at half maximum (FWHM).
(2) An Adaptive Maximum A Posteriori (AMAP) segmentation technique, which models local variations of intensity distributions as slowly varying spatial functions and thus achieves a homogeneous segmentation across cortical and subcortical structures.
(3) A 2nd denoising step using Markov Random Field approach which incorporates spatial prior information of adjacent voxels into the segmentation estimation generated by AMAP.
(4) A Local Adaptive Segmentation (LAS) step, which adjusts the images for white matter (WM) inhomogeneities and varying

| | |
|---|---|
| | gray matter (GM) intensities caused by differing iron content in e.g. cortical and subcortical structures. The LAS step is carried out before the final AMAP segmentation.<br>(5) A Partial Volume Segmentation algorithm that is capable of modeling tissues with intensities between GM and WM, as well as GM and cerebrospinal fluid (CSF) and is applied to the AMAP-generated tissue segments. |
| Normalization | (6) A high-dimensional DARTEL registration of the image to a MNI-template generated from the MRI data of 555 healthy controls in the IXI database (http://www.braindevelopment.org). The registered GM images were multiplied with the Jacobian determinants obtained during registration to produce GM volume maps. |
| Normalization template | MNI template was used |
| Noise and artifact removal | See above (preprocessing steps) |
| Volume censoring | Volume censoring was not performed |

## Statistical modeling & inference

| | |
|---|---|
| Model type and settings | Multivariate modeling was preformed (no classical 1st and 2nd level design). |
| Effect(s) tested | ANOVA/factorial designs were not used. |

Specify type of analysis: ☒ Whole brain ☐ ROI-based ☐ Both

| | |
|---|---|
| Statistic type for inference<br>(See Eklund et al. 2016) | Voxel-wise |
| Correction | Statistical significance of the final prediction set of the SVM classification model was assessed through permutation testing, with α=0.05 and 1000 permutations. |

## Models & analysis

| n/a | Involved in the study |
|---|---|
| ☒ | ☐ Functional and/or effective connectivity |
| ☒ | ☐ Graph analysis |
| ☐ | ☒ Multivariate modeling or predictive analysis |

| | |
|---|---|
| Multivariate modeling and predictive analysis | Following our aims, we employed a nested cross-validated machine learning pipeline (10 permutations and 5 fold on both inner and outer cycle) to evaluate the sensitivity of GM volumetric features at baseline to predict GAF functional response to cognitive training at a single subject level, using a median split strategy. We regressed out the effect of the total GM volumes by entering the values as a covariate. In the inner CV loop, zero-variance features were pruned. Then, a dimensionality reduction procedure was applied through Principal Component Analysis (PCA) in order to minimize the generalization error. PCA was applied to 62 188 voxels contained in GM volume images used in the analysis. PCA projected the image information to a limited number of 80 eigenvariates (80 PCs) in the CV1 training data, that were subsequently scaled (0-1) and then forwarded to the Support Vector Machine (SVM) linear machine-learning algorithm.The optimal hyperparameter C was determined using grid search defined by 11 parameters in the range C = [0.0156 - 16]. Model's performance was measured by Balanced Accuracy (BAC), Sensitivity, Specificity, Positive Predictive Value (PPV), Negative Predictive Value (NPV), and Number Needed to Diagnose (NND). Statistical significance of the final prediction set was assessed through permutation testing, with α=0.05 and 1000 permutations. |
