# Supplementary material for: Multivariate pattern analysis of brain structure predicts functional outcome after auditory-based cognitive training interventions
Source: NPJ Schizophr. 2021 Aug 19;7:40. doi: 10.1038/s41537-021-00165-0 (PMC8376975; doi:10.1038/s41537-021-00165-0)
Supplement: Supplementary file 2 — Supplementary Information [file 41537_2021_165_MOESM2_ESM.pdf]

Supplementary Materials

Long Title: Multivariate pattern analysis of brain structure predicts function outcome after auditory-based cognitive training interventions

Short Title: Brain structure predicts functioning after training

Lana Kambeitz-Ilankovic[1,2], Sophia Vinogradov[3], Julian Wenzel[1,2], Melissa Fisher[3], Shalaila S. Haas[4], Linda Betz[1,] Nora Penzel[1,2], Srikantan Nagarajan[5], Nikolaos Koutsouleris[2], Karuna Subramaniam*[6]

[1] University of Cologne, Faculty of Medicine and University Hospital of Cologne
[2] Department of Psychiatry and Psychotherapy, Ludwig-Maximilian-University, Munich, Germany
[3] Department of Psychiatry, University of Minnesota, USA
[4] Department of Psychiatry, Icahn School of Medicine at Mount Sinai, New York, USA
[5] Department of Radiology and Biomedical Imaging, University of California San Francisco, USA
[6]Department of Psychiatry, University of California San Francisco, USA

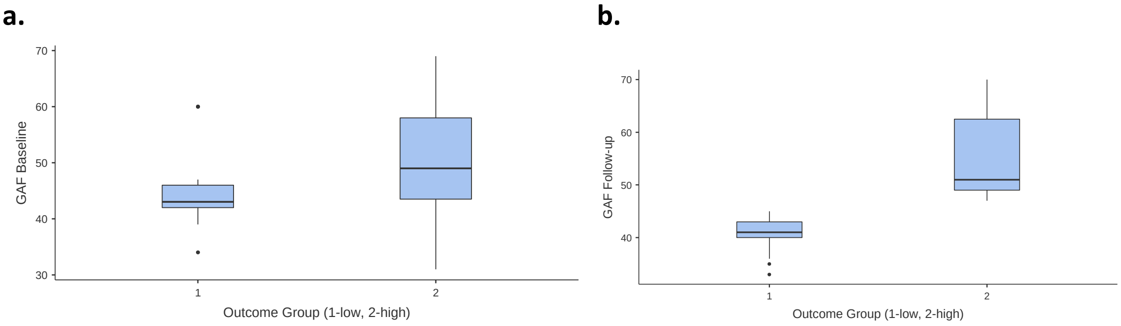

Supplementary Figure 1. Box plots for low and high functioning SCZ patients at **a.** GAF baseline scores and **b.** GAF follow-up scores at the post-training timepoint.

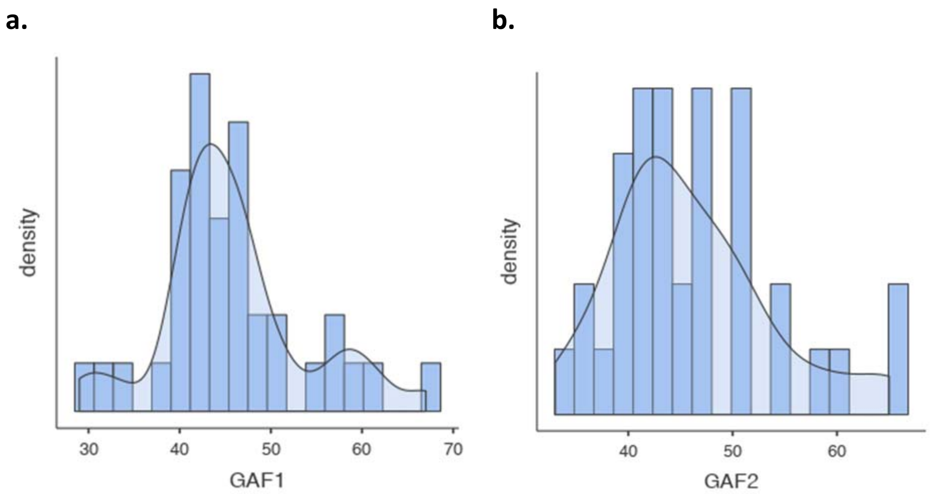

Supplementary Figure 2. The Gaussian distribution of **a.** GAF baseline (GAF1) and **b.** GAF follow-up (GAF2) scores at the post-training timepoint.
